# An enhanced lightweight model for apple leaf disease detection in complex orchard environments

**DOI:** 10.3389/fpls.2025.1545875

**Published:** 2025-03-13

**Authors:** Ge Wang, Wenjie Sang, Fangqian Xu, Yuteng Gao, Yue Han, Qiang Liu

**Affiliations:** ^1^ College of Intelligent Equipment, Shandong University of Science and Technology, Taian, China; ^2^ Technology Department, Shandong Xinhua'an Information Technology Co., Ltd., Qingdao, China

**Keywords:** orchard environments, disease detection, deep learning, ELM-YOLOv8n, DESCS-DH

## Abstract

Automated detection of apple leaf diseases is crucial for predicting and preventing losses and for enhancing apple yields. However, in complex natural environments, factors such as light variations, shading from branches and leaves, and overlapping disease spots often result in reduced accuracy in detecting apple diseases. To address the challenges of detecting small-target diseases on apple leaves in complex backgrounds and difficulty in mobile deployment, we propose an enhanced lightweight model, ELM-YOLOv8n.To mitigate the high consumption of computational resources in real-time deployment of existing models, we integrate the Fasternet Block into the C2f of the backbone network and neck network, effectively reducing the parameter count and the computational load of the model. In order to enhance the network’s anti-interference ability in complex backgrounds and its capacity to differentiate between similar diseases, we incorporate an Efficient Multi-Scale Attention (EMA) within the deep structure of the network for in-depth feature extraction. Additionally, we design a detail-enhanced shared convolutional scaling detection head (DESCS-DH) to enable the model to effectively capture edge information of diseases and address issues such as poor performance in object detection across different scales. Finally, we employ the NWD loss function to replace the CIoU loss function, allowing the model to locate and identify small targets more accurately and further enhance its robustness, thereby facilitating rapid and precise identification of apple leaf diseases. Experimental results demonstrate ELM-YOLOv8n’s effectiveness, achieving 94.0% of F1 value and 96.7% of mAP50 value—a significant improvement over YOLOv8n. Furthermore, the parameter count and computational load are reduced by 44.8% and 39.5%, respectively. The ELM-YOLOv8n model is better suited for deployment on mobile devices while maintaining high accuracy.

## 1 Introduction

As one of the most widely cultivated fruits globally, the annual production of apples significantly influences the agricultural economy and the global food supply chain (Wani and Mishra, 2022). As the largest producer of apples worldwide, China contributes 40.5 million tons annually, representing 46.7% of the global production (Liu et al., 2024b). However, the yield and quality of apples are affected by a variety of diseases, such as Rust, Grey Spot, Powdery Mildew, and Scab, which not only reduce the quality of fruits, but also directly lead to a decrease in yield. Therefore, accurate and timely identification and detection of leaf diseases is crucial during apple cultivation. This can assist farmers in more effectively controlling the spread of diseases, thereby maintaining apple quality and production levels, and ultimately achieving both economic and environmental benefits. Traditional identification of crop diseases primarily depends on the expertise of agricultural specialists and visual assessment. This method is inefficient and limited by the expert’s knowledge and experience, which is prone to misjudgment and omission. With the development of computer technology, machine learning has started to find application in the recognition of crop diseases (Ramesh et al., 2018). However, traditional feature extraction algorithms usually need to rely on manual design and domain expertise to select and construct features. Compared to traditional machine learning methods, deep learning methods can automatically learn and extract features, reducing the workload of manual feature engineering and exhibiting better performance when dealing with large-scale data. In 2020, Li et al. (2020) introduced a finely-tuned GoogLeNet model, which was fine-tuned to improve over the state-of-the-art method by 6.22%.In 2021, Soujanya and Jabez (2021) proposed a technique based on improved AlexNet that can successfully classify 38 different classes of healthy and diseased plants, and the best accuracy of this method is 96.5%.In 2022, Wei et al. (2022) proposed a multi-scale feature fusion based network model for accurate identification and classification of crop pests and diseases, the classification accuracy reached 98.2%.In 2023, Islam et al. (2023) used ResNet50 migration learning model as the core for distinguishing between healthy and infected leaves and classifying current disease types, providing an even higher accuracy of 98.98%. In 2024, Faisal et al. (2024) proposed a method based on deep CNN architecture to recognize and classify cotton weeds efficiently and the proposed model achieved 98.3% accuracy which is better than other models. Although these studies have achieved satisfactory accuracy rates when dealing with the task of classifying a single disease image with a simple background, however, the performance may still be insufficient in real-world applications when faced with more complex and varied scenarios, as well as in mobile deployments.

Faced with real-time detection in complex environments, object detection algorithms (Ren et al., 2016; Liu et al., 2016; Redmon, 2016) show greater advantages. Sun et al. (2021) proposed a lightweight CNN model, MEAN-SSD, which can be deployed on mobile devices for real-time detection of apple leaf diseases. Khan et al. (2022) proposed a two-stage real-time detection system for apple leaf diseases based on Xception and Faster-RCNN-based two-stage real-time apple disease detection system, which achieved an overall classification accuracy of 88%. Zhang et al. (2023b) designed BCTNet network for accurate apple leaf disease detection, which solved the problems of unconstrained environmental factor interference and low detection accuracy caused by significant changes in the target scale of apple leaf disease detection. Lv and Su (2024) proposed YOLOV5-CBAM-C3TR for apple leaf disease detection, which showed strong recognition ability in identifying similar diseases, and is expected to promote the further development of disease detection technology. Chang and Lai (2024) integrated a lightweight convolutional neural network architecture, RegNetY-400MF, with a transfer learning technique, which not only improves the accuracy of potato leaf disease detection, but also reduces the computational and storage requirements. Liu et al. (2024a) proposed MCDCNet to extract more reliable apple leaf disease features with various scales and geometries, which effectively improved the discriminative ability of the network. However, the reduction of model parameters and computational load may lead to the decrease of accuracy, how to strike a balance between lightness and accuracy is a hot research topic nowadays (Yan et al., 2024).

In summary, although the crop disease recognition method based on Convolutional Neural Networks (CNN) solves the problem of inefficiency of traditional machine vision recognition, there are still problems such as high model complexity and low accuracy of small target disease recognition under complex background. To address these challenges, this study introduces a lightweight detection model for identifying apple leaf diseases against complex backgrounds. First, the Fasternet module is integrated to decrease the number of parameters and computational load of the model; second, an Efficient Multi-Scale Attention (EMA) is incorporated to improve the feature extraction ability of the network for small target diseases as well as the differentiation ability for similar diseases; then, the DESCS-DH detection head is designed to optimize the model’s effectiveness in the face of multi-scale target detection. Finally, the NWD loss function is employed to improve the model’s precision in localizing and identifying small targets, thereby enhancing the model’s robustness and facilitating rapid and accurate identification of apple leaf diseases.

## 2 Related works

With the development of artificial intelligence technology, machine learning and deep learning have been widely used in the field of automatic crop disease detection. The following is a brief overview of these two methods.

### 2.1 Machine learning methods

This approach first requires the extraction of disease features from collected plant images, which are subsequently used to train machine learning models that typically require more domain knowledge and human intervention to select and optimize features. In 2022, Harakannanavar et al. (2022) developed an algorithm based on machine learning and image processing to automatically detect tomato leaf disease. SVM, KNN, and CNN were used to classify features, and the accuracy of the three methods used was 88%, 97%, and 99.6%, respectively. In 2023, Gaikwad and Musande (Gaikwad and Musande, 2023) developed an advanced crop disease prediction technique using cetalatran-optimization algorithm, deep KNN, and relief algorithm, the accuracy reached 91.879%. Ahmed and Yadav (2023) developed a crop disease prediction model using back propagation ANN, SVM, GLCM, and k-mean algorithm, the accuracy reached 99%.

### 2.2 Deep learning methods

Deep learning methods, such as convolutional neural networks (CNN), can learn features directly from the original image without the need for a complex feature extraction process. At present, the research on deep learning in the field of plant disease diagnosis is developing rapidly, which provides strong technical support for agricultural production. Many researchers use famous CNN architectures such as AlexNet, VGG, GoogLeNet, and ResNet for disease classification. Anim-Ayeko et al. (2023) proposed a ResNet9 model that detects the blight disease in both potato and tomato leaf images for farmers to leverage, the model achieved 99.25% accuracy. Reddy et al. (2023) proposed a customized PDICNet model for crop disease identification and classification, with an accuracy and F1-score of 99.73% and 99.78%, respectively, for PlantVillage dataset. Current models based on disease classification have reached a high accuracy.

To further localize where the disease is located, two-stage detection algorithms and single-stage detection algorithms have been applied to disease detection, such as Faster R-CNN, YOLO, and SSD. We chose advanced object detection models for apple leaf disease detection and analyzed them in comparison with our model in comprehensive aspects. As shown in **Table 1**, for dataset selection, we synthesized the two most commonly used publicly available apple disease datasets to ensure diversity of data sources. In terms of disease types, we chose a wider range of disease types, selecting six of the most common ones. In terms of research methodology, we not only pay attention to the improvement of model performance, but also focus on the resource consumption of the model. In addition to improving the traditional feature extraction module, we also proposed a Detail-Enhanced Shared Convolutional Scaling Detection Head, so that the model can more accurately capture the subtle features of the disease when facing multi-scale targets. The model we developed not only achieves excellent accuracy, with a mAP50 of 96.7%, but also optimizes the number of model parameters and the amount of computation, effectively alleviating the problem of resource consumption. In terms of the adaptability of application scenarios, our model is able to cope with complex field environments, showing good practicality and flexibility.

**Table 1:** Comparison of object detection models for apple leaf disease detection.

Model	Research Method	Data Source	Disease Type	Results
MEAN-SSD (Sun et al., 2021)	A new apple leaf disease detection model MEAN-SSD was constructed using MEAN block and Apple-Inception module.	Self-built datasets AppleDisease5	Five categories.	Achieved 83.12% mAP and 12.53 FPS detection performance.
FPN-ISResNet-Faster RCNN (Hou et al., 2023)	This study proposes a deep learning model called the feature pyramid networks (FPNs) –inception squeeze-and-excitation ResNet (ISResNet)–Faster RCNN.	FGVC8 and AI Challenger 2018	Three categories.	The mAP50 is 93.68%.
YOLO-Leaf (Li et al., 2024)	Feature extraction using DSConv, enhancement of attention mechanism using BiFormer, introduction of IF-CIoU for improved bounding box regression.	FGVC7 and FGVC8	Categories four and five.	The mAP50 scores reached 93.88% and 95.69%, respectively.
YOLOv8n–GGi (Gao et al., 2024)	GhostConv replaces the traditional Conv layer, replaces part of the C2f structure with C3Ghost, integrates a Global Attention Mechanism (GAM), and incorporates an improved BiFPN.	AppleLeaf9	Four categories.	The mAP50 is 86.9%, GFLOPs is 5.5, Parameters is 1.7M.
ELM- YOLOv8n(Ours)	Design lightweight module c2f-faster, extract subtle features using the EMA attention mechanism, propose Detail-Enhanced Shared Convolutional Scaling Detection Head, and improvement of loss function.	FGVC8 and AppleLeaf9	Six categories.	The mAP50 is 96.7%, GFLOPs is 4.9, Parameters is 1.6M.

## 3 Materials and methods

### 3.1 Dataset construction

The experimental datasets were sourced from the publicly available datasets plant-pathology-2021-fgvc8 and AppleLeaf9.Both datasets contain a large number of high-quality images of apple leaf diseases, from which six leaf diseases were selected for labeling, namely Rust, Scab, Grey Spot, Frog Eye Leaf Spot, Powdery Mildew, and Alternaria Blotch, with more than 90% of the images collected in the orchard environments. These six diseases are prevalent on apple leaves, resulting in significant detrimental effects on agricultural productivity, and they are representative in terms of symptom presentation, covering different lesion types such as spotting, powdering, and rusting. There are differences in the difficulty of recognizing different diseases. For instance, rust is typically more easily recognizable by the model because of its distinctive color and shape feature, whereas Alternaria Blotch and Frog Eye Leaf Spot impose greater demands on the model’s discriminatory capabilities due to their more similar lesion characteristics.

**Figure 1:**
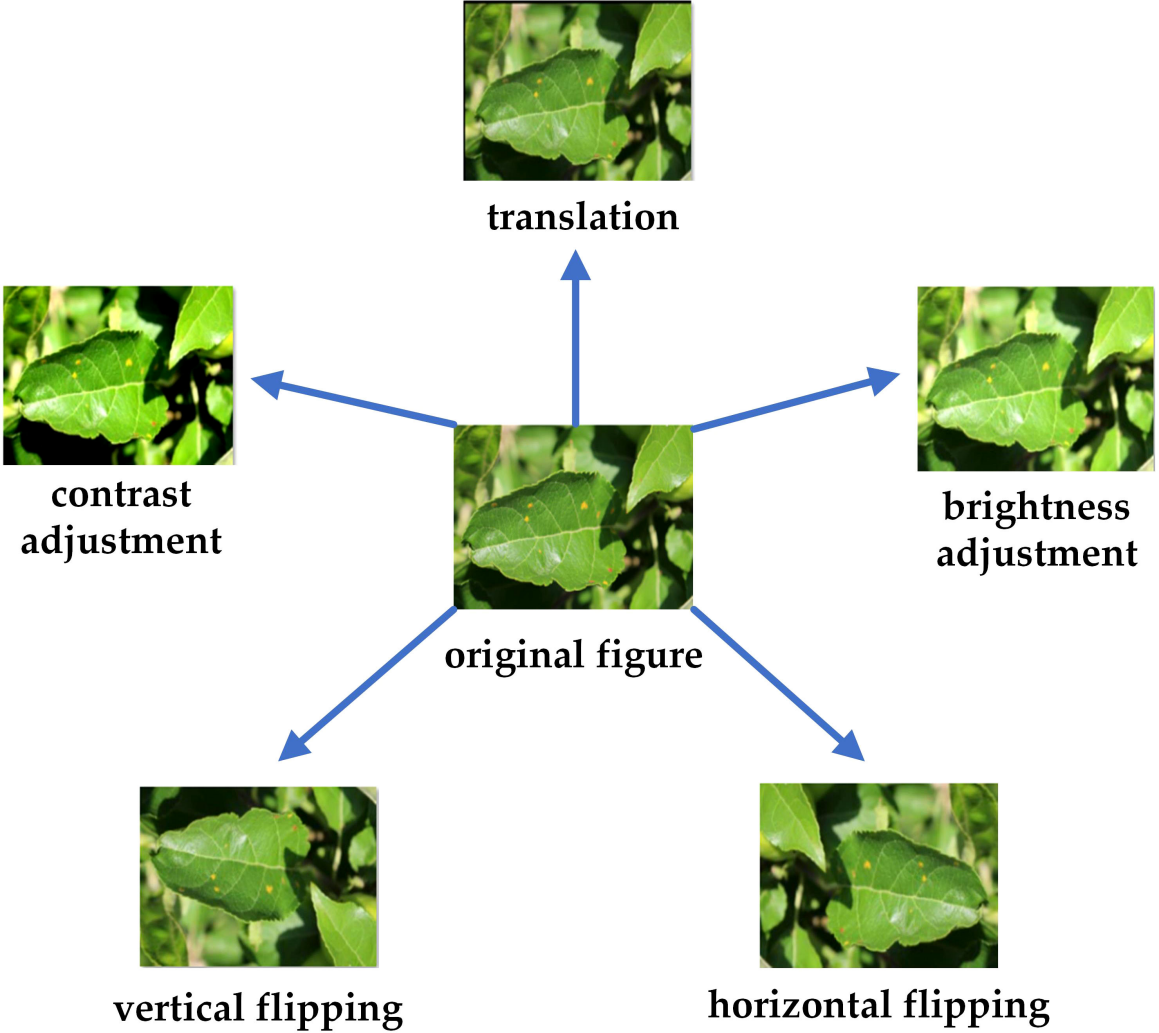
Data enhancement methods.

A total of 2,203 images were labeled using an online platform, Make Sense. The small target box is used to label Rust, Grey Spot, Frog Eye Leaf Spot, and Alternaria Blotch. For Scab and Powdery Mildew, which develop throughout the leaf, the entire leaf is labeled. The dataset was augmented through data enhancement techniques. To prevent both the original and enhanced images from appearing simultaneously in the training and validation sets, the original images were initially divided into training, validation, and test sets in a ratio of about 8:1:1.Our model requires a large amount of data to capture the subtle differences between different diseases, and this ratio ensures that the model has enough data for training and the right amount of data for validation and testing. Subsequently, the images and labels were augmented using five techniques: horizontal flipping, vertical flipping, translation, contrast adjustment, and brightness adjustment, the new dataset obtained is named Apple-Leaf1.An example of the enhancement methods is illustrated in **Figure 1**. The numbers and sources of the images are presented in **Table 2**.

**Table 2:** Number and source of images in the Apple-Leaf1 dataset.

Disease Name	Image Source	Original Quantity	Enhanced Training Set	Enhanced Validation Set	Enhanced Test Set
Rust	2021-fgvc8	521	2496	282	348
Scab	2021-fgvc8	345	1620	234	216
Grey Spot	2021-fgvc8	316	1560	162	174
Frog eye leaf spot	2021-fgvc8	365	1794	192	204
Powdery Mildew	AppleLeaf9	348	1692	210	186
Alternaria Blotch	AppleLeaf9	308	1404	240	204
Total		2203	10566	1320	1332

### 3.2 Methodology

#### 3.2.1 Fasternet module

**Figure 2:**
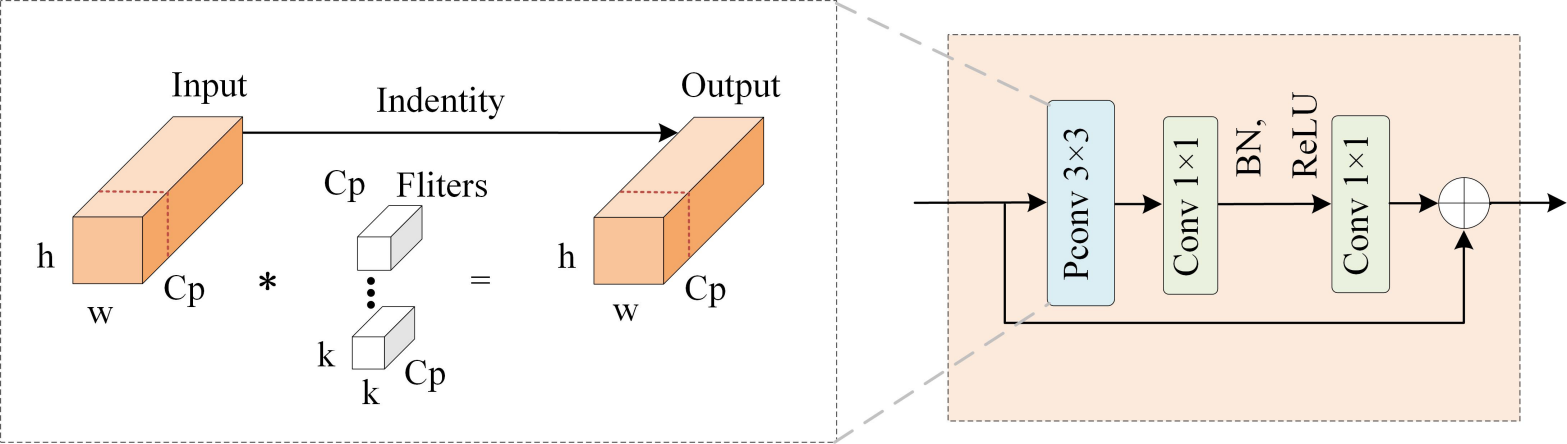
Fasternet Block. The "*" represents Convolution.

In order to design lighter and faster networks, many researchers have generally focused on reducing the total amount of floating-point operations (FLOPs) (Menghani, 2023; Zeng et al., 2022; Zhong et al., 2022). However, merely reducing FLOPs does not invariably result in the desired reduction in delay. The underlying reason lies in the inefficiency of the actual floating-point operations per second (FLOPS) performed. Chen et al. (2023) designed a new convolution (PConv), which is able to significantly reduce unnecessary computations and memory accesses while extracting spatial features through a subtle design, thus significantly improving the computational efficiency. Based on this, the FasterNet family was further developed, as shown in **Figure 2**, which is a new set of neural network architectures.

In this study, we integrate the Faster Block into the backbone and neck to obtain C2f-Faster, which reduces the number of parameters and computational load of the model. The Faster Block is designed to fully incorporate the high efficiency of PConv.The FLOPs of PConv, as shown in Equation 1, is 1/16 of the conventional convolution.


(1) $$h\times w\times k^{2}\times c_{\text{p}}^{2}$$

And the memory access of PConv, as shown in Equation 2, is 1/4 of the conventional convolution.


(2) $$h\times w\times 2c_{p}+k^{2}\times c_{p}^{2}\approx h\times w\times 2c_{p}$$

The Faster Block achieves a significant reduction in computational complexity and memory access by introducing PConv, which applies regular convolution operations to a portion of the input channel while leaving the remaining channels unchanged in their original state. Although only a portion of the input channel is computed, the preserved channel remains useful in subsequent PWConv layers, allowing feature information to flow through all channels. This design enables the Faster Block to operate rapidly on various hardware platforms, including mobile devices, while maintaining high detection accuracy.

#### 3.2.2 EMA module

**Figure 3:**
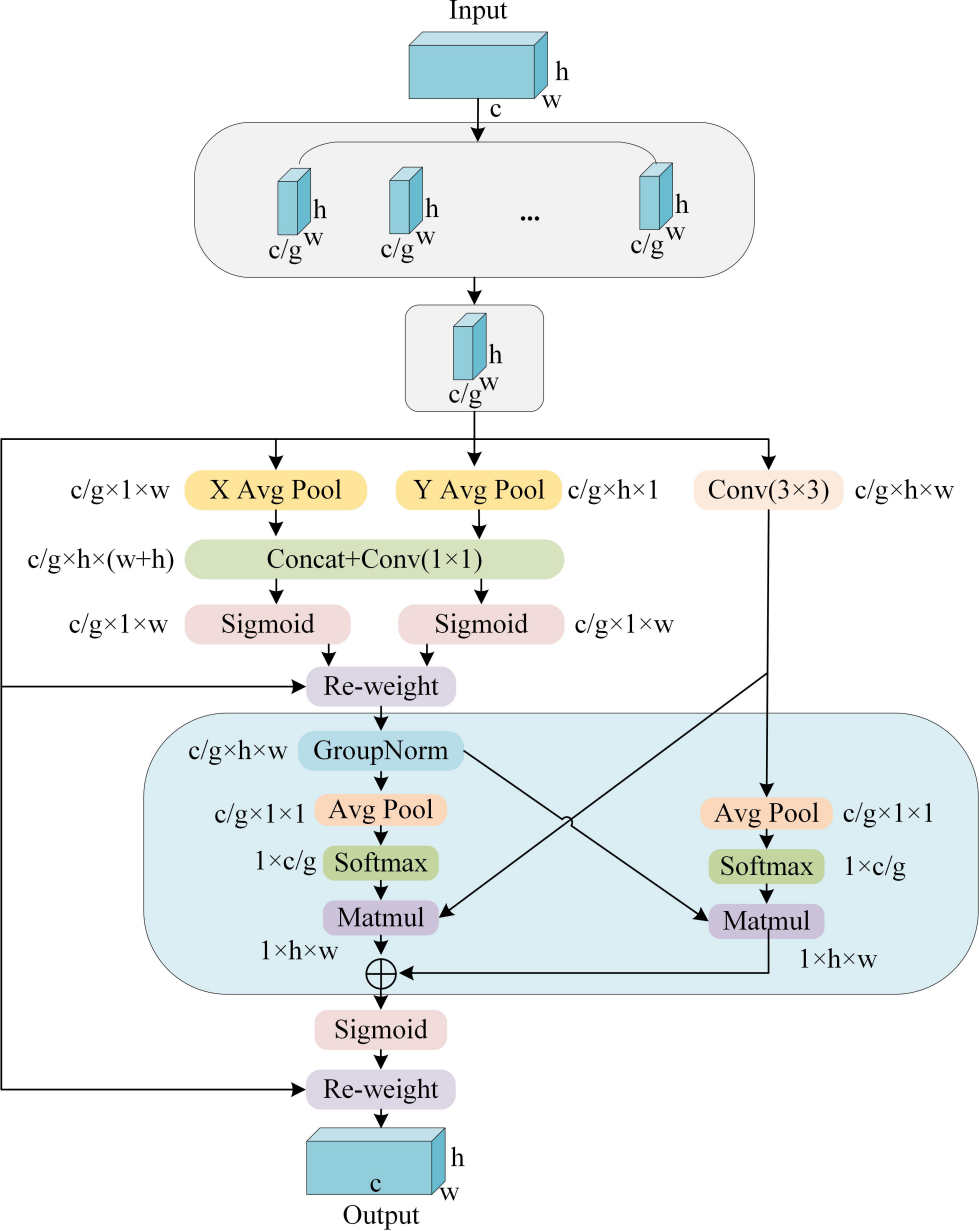
EMA module.

In computer vision tasks, channel or spatial attention mechanisms effectively extract local features of an object (Sun et al., 2022; Wang et al., 2024b; Zheng et al., 2023). Ouyang et al. (2023) proposed the Efficient Multi-Scale Attention, which pursues a balance between computational efficiency and information retention by adopting an innovative approach: reconstructing some channels into batch dimensions and subdividing the channel dimensions into multiple sub-feature groups. This ensures a uniform distribution of spatial semantic features within each sub-feature group, thereby optimizing feature expression and processing without adding computational burden. As illustrated in **Figure 3**, the EMA module first divides the input into multiple grouped feature maps, which are then processed through three parallel path branches: two perform one-dimensional global pooling, and the third conducts feature extraction via 3x3 convolution. Ultimately, the output feature maps within each group are aggregated by computing the two generated spatial attention weights and then processed using a Sigmoid function to capture pairwise pixel relationships and emphasize the global contextual information of all pixels.

**Figure 4:**
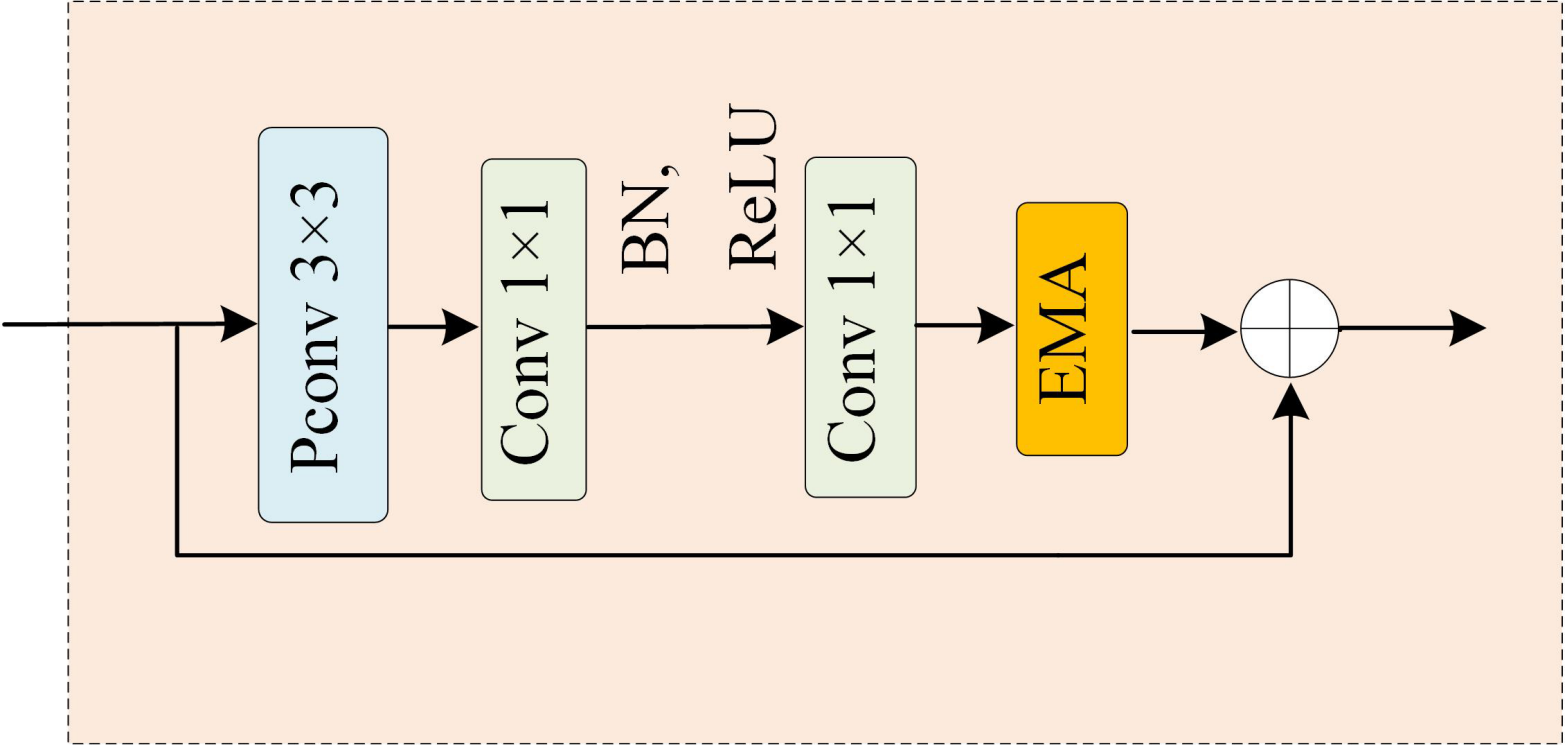
Architecture for Fasternet-EMA.

The onset sites of apple leaf diseases typically appear as small spots on the leaves, particularly during the early stages of infection. However, these small lesions are difficult to detect due to the complex background. Furthermore, the initial symptoms of several apple leaf diseases, such as Alternaria Blotch, Grey Spot, and Frog Eye Leaf Spot, are remarkably similar, making them challenging to distinguish. To address the issue of insufficient feature extraction for small target disease against a complex background, the EMA mechanism is integrated into the deep layers of the network’s backbone, as illustrated in **Figure 4**. Feature extraction is conducted in depth following Pconv and two 1×1 convolution operations, enabling the model to focus more on disease details and thereby enhancing recognition accuracy.

#### 3.2.3 DESCS-DH (detail-enhanced shared convolutional scaling detection head)

Although YOLOv8 performs well on many tasks, there is still room for improvement in the design of the detection head. The first is the large number of parameters, the original detection head employs one 1×1 and two 3×3 convolutions for feature extraction before predicting the object class and positional offset within each bounding box, which inevitably results in a considerable parameter increase; second is that the use of a normal convolution does not capture the detailed information of the image very well during feature extraction, which may lead to important information loss; and lastly, the reliance on predefined anchors may cause the model to perform poorly in detecting targets of different scales and proportions, as the anchors may not be well adapted to the shapes and sizes of all objects. To address these challenges, we design a detail-enhanced shared convolutional scaling detection head named DESCS-DH.

**Figure 5:**
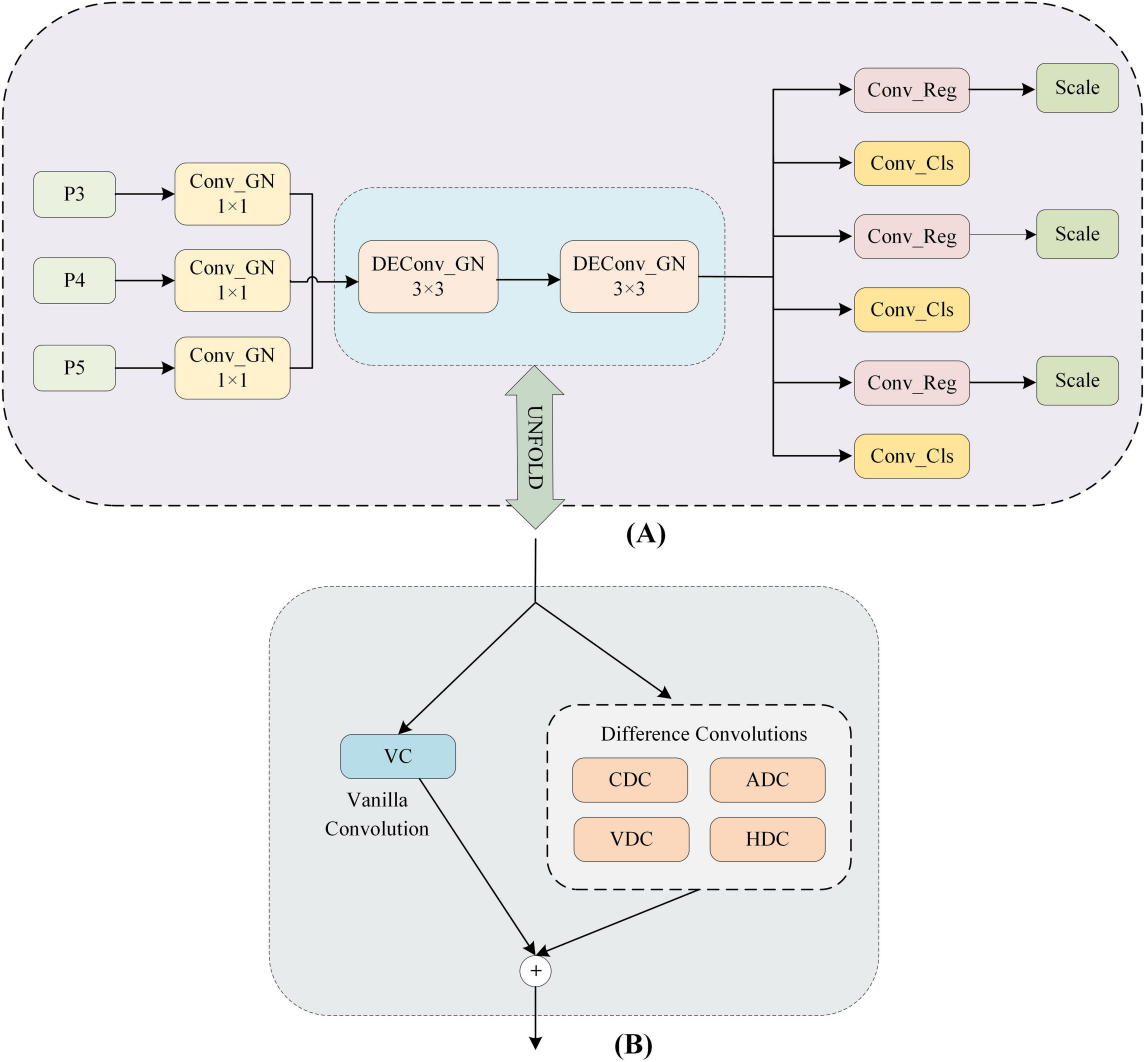
The structure of DESCS-DH. **(A)** DESCS-DH **(B)** DEConv.

DESCS-DH is an efficient and lightweight detection head, which can maintain high accuracy with reduced number of parameters and computation. As shown in **Figure 5**, firstly, DESCS-DH significantly reduces the number of parameters by fusing P3, P4, and P5 feature maps at different scales through a shared convolutional layer for feature interaction and parameter sharing. Secondly, it uses detail-enhanced convolution to improve the ability of the detection head to capture image details. DEConv significantly enhances the model’s representation and generalization performance by incorporating *a priori* information into the ordinary convolutional layers. In addition, through the application of reparameterization techniques, DEConv can be equivalently converted to ordinary convolution, a process that does not require the addition of extra parameters or computational cost, thus effectively improving performance while keeping the model lightweight. Finally, DESCS-DH can adaptively modify its internal parameters based on the dimensions of the input image and feature map, so that when facing multi-scale targets, each detector head can obtain the optimal working parameters, which enables it to maintain stable performance in a variety of complex environments, thereby enhancing the robustness of the entire model and its detection efficiency.

#### 3.2.4 NWD loss function

In the field of object detection, Intersection over Union (IoU) has long been a standard metric for measuring the accuracy of bounding boxes (Peng and Yu, 2021; Zhang et al., 2022; Tian et al., 2022). However, for the detection of tiny objects, the performance of IoU is not satisfactory, which is mainly due to the small overlapping area of the bounding box of tiny objects, resulting in a low value of IoU, which is difficult to accurately reflect the actual detection accuracy. To solve this problem, Wang et al. (2021) proposed a novel metric based on the Wasserstein distance, aiming to evaluate the similarity of the bounding boxes of tiny objects more effectively.

Specifically, the method first models the shape and position information of the bounding box as a two dimensional Gaussian distribution. This modeling approach captures the characteristics of the bounding box in more detail, especially for objects with irregular shapes or small sizes. Next, the Normalized Wasserstein Distance (NWD) is introduced to quantify the similarity between these Gaussian distributions. An additional significant benefit of the NWD is its insensitivity to object scale, enabling more precise localization for tiny objects. In addition, the application of NWD is not limited to the label assignment stage; it can also replace the IoU in the non-maximal suppression (NMS) process and be used in the regression loss function, thereby improving the performance of tiny object detection in general. The computational procedure is as follows, after modeling the bounding box as a two dimensional (2D) Gaussian distribution, the distribution distance is calculated using the Wasserstein distance metric. For two dimensional Gaussian distribution, u1 and u2 as shown in Equation 3:


(3) $$\mu_{1}=N({\text{m}}_{1},{\text{Σ}}_{1}),\mu_{2}=N({\text{m}}_{2},{\text{Σ}}_{2})$$

Define the distance between them as Equation 4:


(4) $$W_{2}^{2}(\mu_{1},\mu_{2})=\|m_{1}-m_{2}{\|}_{2}^{2}+Tr({\text{Σ}}_{1}+{\text{Σ}}_{2}-2{\left({\text{Σ}}_{2}^{1/2}{\text{Σ}}_{1}{\text{Σ}}_{2}^{1/2}\right)}^{1/2})$$

The simplified formula is shown in Equation 5:


(5) $$W_{2}^{2}(\mu_{1},\mu_{2})=\|m_{1}-m_{2}{\|}_{2}^{2}+\|{\text{Σ}}_{1}^{1/2}-{\text{Σ}}_{2}^{1/2}\|{\;}_{F}^{2}$$

Furthermore, for Gaussian distributions $N_{a}$ and $N_{b}$ , which are derived from bounding boxes $\text{A }=\;(\text{c}{\text{x}}_{\text{a}},\text{ c}{\text{y}}_{\text{a}},\;{\text{w}}_{\text{a}},\;{\text{h}}_{\text{a}})$ and $\text{B }=\;(\text{c}{\text{x}}_{\text{b}},\text{ c}{\text{y}}_{\text{b}},\;{\text{w}}_{\text{b}},\;{\text{h}}_{\text{b}})$ , Equation 5 can be simplified to Equation 6:


(6) $$W_{2}^{2}(N_{a},N_{b})=\|{\left({\left[cx_{a},cy_{a},\frac{w_{a}}{2},\frac{h_{a}}{2}\right]}^{\text{T}},{\left[cx_{b},cy_{b},\frac{w_{b}}{2}.\frac{h_{b}}{2}\right]}^{\text{T}})\right\|}_{2}^{2}$$

However, the numerical range of $W_{2}^{2}(N_{a},\;N_{b})$ is not suitable for direct use as a similarity metric. Consequently, its exponential form is adopted for normalization, yielding a new metric termed the Normalized Wasserstein Distance (NWD), as shown in Equation 7:


(7) $$NWD(N_{a},N_{b})=\text{exp }(-\frac{\sqrt{W_{2}^{2}(N_{a},\;N_{b})}}{C})$$

In the detection and localization of small target diseases, the limited pixel occupation in the image results in scarce semantic information, making it challenging for the network to extract features that are sufficiently effective for precise target categorization and localization. The NWD loss function exhibits a unique sensitivity to both the size of the target and the distances between targets. Traditional loss functions may not fully consider these factors. For apple leaf disease detection, accurately locating and distinguishing spots of different sizes and location relationships is crucial for enhancing detection accuracy. The NWD loss function facilitates the model’s focus on features pertinent to disease detection during training, owing to its sensitivity to target size and inter-target distances.

#### 3.2.5 ELM-YOLOv8n network architecture

**Figure 6:**
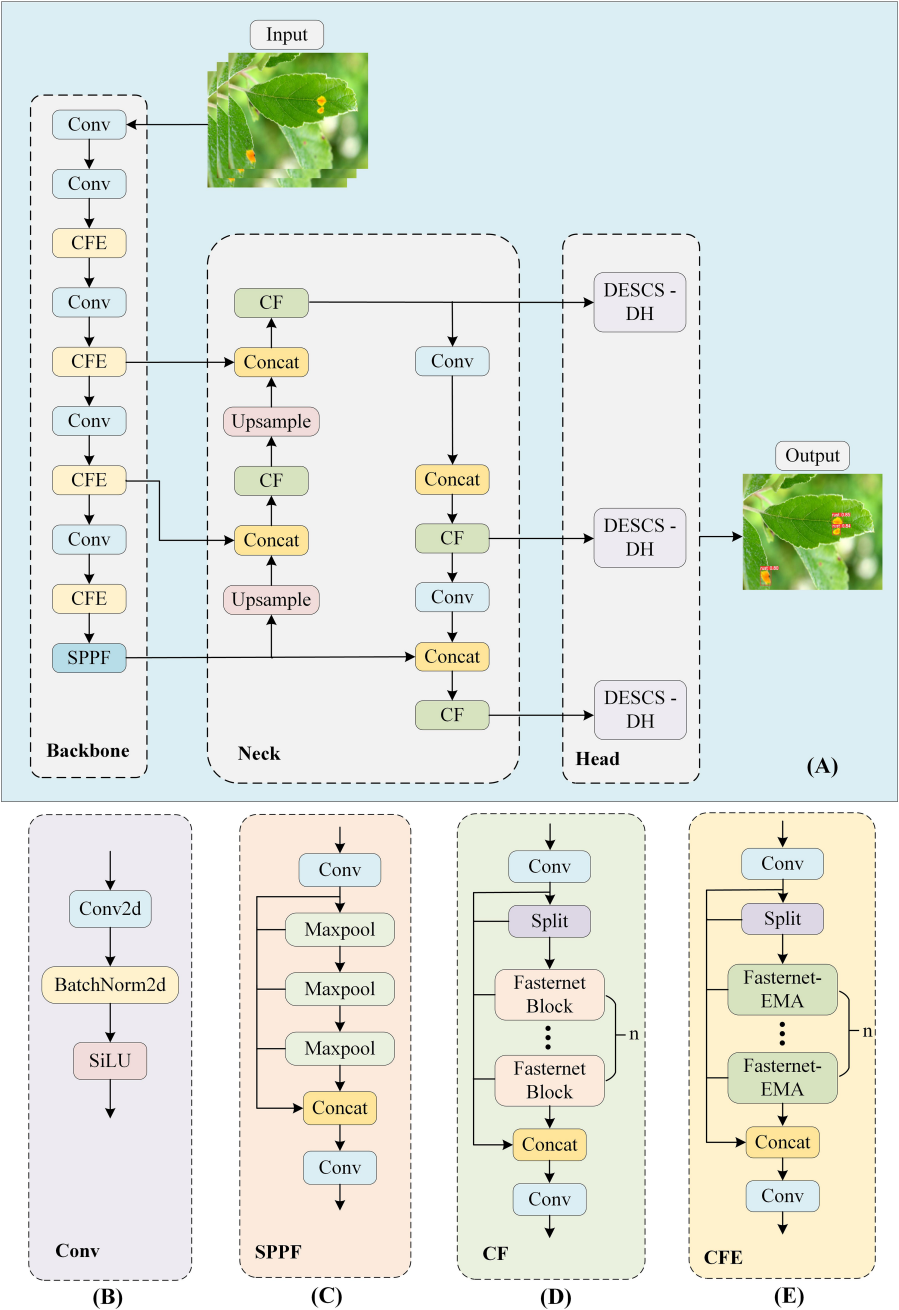
ELM-YOLOv8n overall framework and its component modules **(A)** ELM-YOLOv8n. **(B)** Conv. **(C)** SPPF. **(D)** C2f-Faster. **(E)** C2f-Faster-EMA.

YOLOv8, an advanced object detection algorithm, effectively strikes a balance between detection accuracy and speed (Wan et al., 2024; Zhu et al., 2024). Consequently, in this study, we selected and enhanced the YOLOv8n model to develop a new network architecture termed ELM-YOLOv8n.The YOLOv8 architecture comprises four components: Input, Backbone, Neck, and Head. The Backbone extracts features from the input image data using CSPDarknet53 to convert raw pixels into high-level semantic features. The Neck serves as the middle layer of the network structure and fuses the features of different levels through techniques such as Feature Pyramid Network (FPN).The Head is the output layer of the network, which predicts the information of the target’s category, location, and confidence level based on the fused features. As shown in **Figure 6**, the enhanced lightweight model ELM-YOLOv8n is designed in this research to realize the model to reduce the consumption of computational resources while maintaining high detection performance. The C2f module in the original YOLOv8 consumes a significant portion of the network’s parameters. By substituting the Bottleneck with the Fasternet Block, we integrate the C2f-Faster structure, which effectively diminishes the parameter count and model complexity. The EMA attention is incorporated into the deepest layer of the Fasternet Block in Backbone, and after Pconv and two 1×1 convolutional operations, the deep subtle features are extracted. The Head employs the DESCS-DH, addressing the issues of high parameter count and inadequate detail capture in the original detector head, as well as inconsistencies in performance across varying target sizes. Lastly, the NWD loss function for small target detection is utilized in place of the CIOU loss function to enhance the model’s accuracy in localizing small targets.

### 3.3 Experimental environment

#### 3.3.1 Experimental platform

The experiment was conducted using a 64-bit Windows 11 operating system, with the PyTorch framework, PyCharm IDE, and Python programming language employed for model training. To enhance data diversity and mitigate overfitting, the mosaic augmentation technique was applied during the training phase. The detailed experimental environment and some experimental parameters are presented in **Tables 3**, **4**, respectively.

**Table 3:** Experimental environment.

Environment	Configuration
Operating system	Windows 11
CPU	AMD Ryzen 7 7735H
GPU	NVIDIA GeForce RTX 4060
RAM	16GB
Programming language	Python 3.8.19
Deep learning framework	Pytorch 2.2.1
CUDA	CUDA version12.1

**Table 4:** Experimental parameters.

Parameters	Setup
Input image size	640 × 640
Batch size	16
Workers	8
Optimizer	SGD
Learning rate	0.01
Epochs	100

#### 3.3.2 Evaluation metrics

The model is evaluated on two dimensions: performance and complexity. Performance metrics include precision (P), which measures the proportion of positive samples detected by the model that are actually positive; recall (R), which refers to the proportion of samples that are actually in the positive category that are correctly predicted by the model to be in the positive category; F1 score, which is the harmonic mean of precision and recall; average precision (AP), which is a combination of precision and recall and is obtained by plotting a precision-recall curve and calculating its area; and mean average precision in multiple categories(mAP), which calculates the AP for each category and averages the values. The specific calculation formulas are shown in Equations 8–12:


(8) $$\text{P}=\frac{\text{TP}}{\text{TP}+\text{FP}}\times 100\%$$

(9) $$\text{R}=\frac{\text{TP}}{\text{TP}+\text{FN}}\times 100\%$$

(10) $$F1=\frac{2\times P\times R}{P+R}\times 100\%$$

(11) $$\text{AP}=\int_{0}^{1}\text{P }(\text{R})\text{ dR}\times 100\%$$

(12) $$\text{mAP}=\frac{1}{N}\sum_{\text{k}=1}^{\text{k}=\text{N}}{\text{AP}}_{\text{k}}\times 100\%$$

In complexity evaluation, three main indicators are considered: the number of model parameters(Params), Giga Floating-point Operations Per Second (GFLOPs), and the model size. The number of parameters of the model is the total number of all trainable parameters in the model, which are optimized during training to reduce prediction errors.The parameter count serves as a crucial indicator of a model’s complexity. GFLOPs is used to measure the amount of model computation. Model size denotes the memory space required for storing the model on disk and aids in evaluating its deployment viability in environments with limited resources.

## 4 Results and analyses

### 4.1 Effect of incorporating different attention mechanisms into the backbone C2f on model performance

Incorporating different attention mechanisms into the deepest layer of the Fasternet Block in the backbone network, the attention mechanisms together with the lightweight module resulted in a reduction in the number of parameters and computation of the whole model. As shown in **Table 5**, the CF-LSKA has the highest precision of 94.6%. In the F1 score, a composite metric, both CF-TripleAttention and CF-EMA achieve 92.5%, indicating that they strike a good balance between precision and recall. On the key metrics of mAP50 and mAP50-95, CF-EMA performs well with 96.3% and 66.5%, respectively. Finally, CF-EMA shows excellent efficiency in terms of parameter count and computation, with its parameter count of only 2.65M and its GFLOPs remaining at 7.1, which means that it maintains high performance with high computational efficiency and low resource consumption.

**Table 5:** Effect of different attention mechanisms on model performance.

Attention	P%	R%	F1%	mAP50%	mAP50-95%	Params/M	GFLOPs
CF-LSKA	94.6	90.0	92.2	96.1	66.3	2.68	7.1
CF-CAFM	92.4	91.9	92.1	95.8	66.2	2.86	8.1
CF-SEAttention	93.1	91.3	92.2	95.8	65.3	2.65	7.0
CF-TripleAttention	94.5	90.5	92.5	96.2	66.4	2.65	7.0
CF-CAA	93.0	91.3	92.1	95.9	65.7	2.71	7.2
CF-MLCA	92.5	91.2	91.9	95.9	66.0	2.65	7.0
CF-EMA	94.0	91.0	92.5	96.3	66.5	2.65	7.1

### 4.2 Effect of different detection heads on model performance

Among the four different detection heads, with the exception of the Aux detection head (Wang et al., 2023), the other three detection heads reduce the number of parameters and computational complexity of the model. Specifically, LADH (Zhang et al., 2023a) has the lowest computational complexity, but this is accompanied by lower accuracy. As presented in **Table 6**, LADH has relatively low F1 scores and mAP50 values. The Aux detection head has the highest accuracy. However, on the whole, the DESCS-DH has relatively high F1 score and mAP50 value, and also has a low number of parameters and computational complexity, thus achieving a balance between performance and computational resources.

**Table 6:** Effect of different detection heads on model performance.

Detection Head	P%	R%	F1%	mAP50%	mAP50-95%	Params/M	GFLOPs
LADH	92.4	90.9	91.6	95.3	66.3	2.36	5.7
Auxhead	94.0	89.6	91.7	95.2	66.3	3.76	11.2
SEAMHead	93.1	92.0	92.5	96.0	66.5	2.82	7.0
DESCS-DH	93.7	91.3	92.5	96.3	66.6	2.36	6.5

### 4.3 Performance comparison of different loss functions

**Figure 7:**
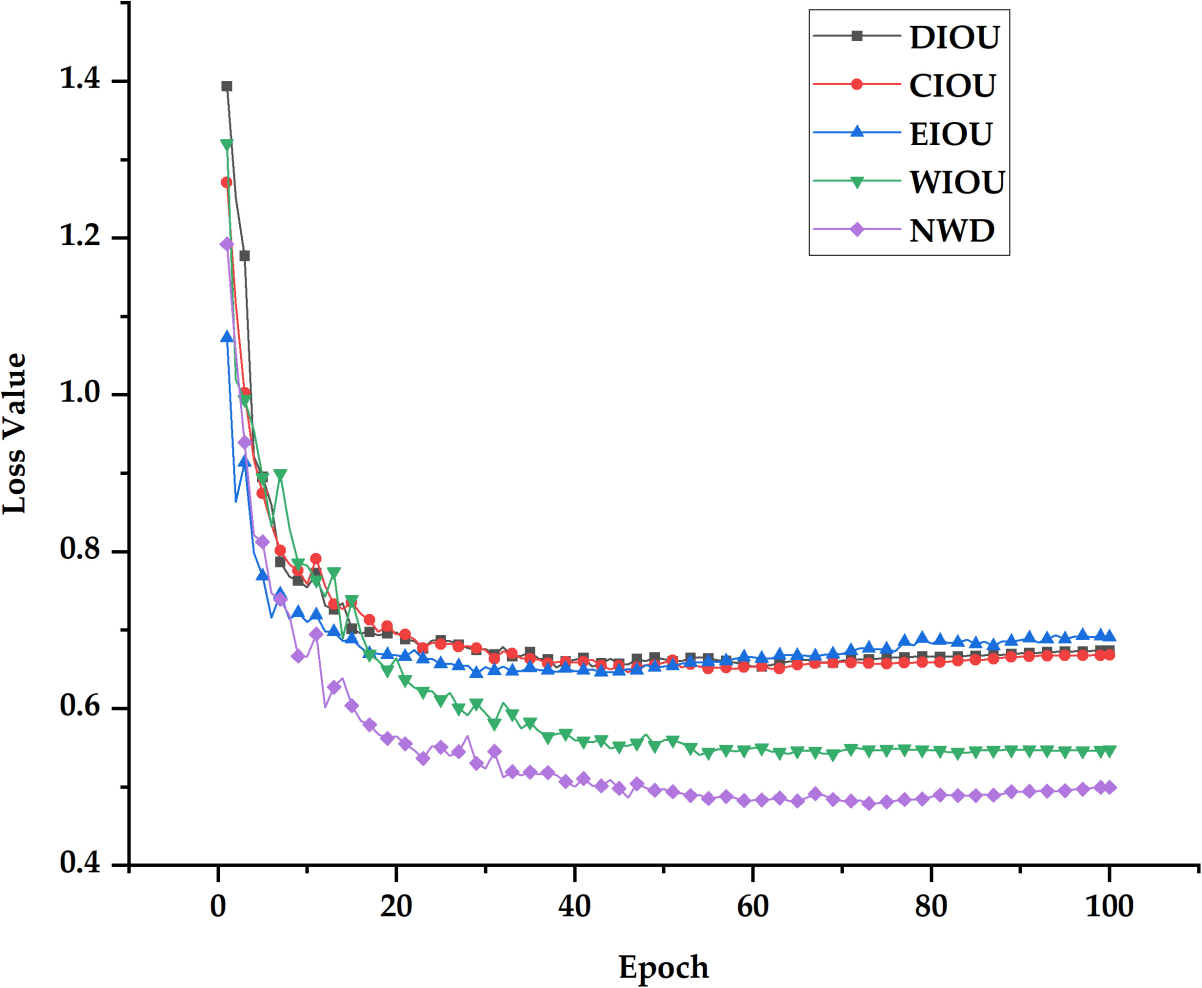
Change in loss value.

Model training and validation were performed using different loss functions. As presented in **Table 7**, the NWD loss function yields the highest precision, F1, and mAP50, with respective values of 94.1%, 93.5%, and 96.6%. As shown in **Figure 7**, all five loss functions significantly decreased the model’s boundary regression loss throughout the network’s training process. However, the DIOU, CIOU and EIOU resulted in relatively high loss value, whereas the NWD loss function achieved the lowest loss value.

**Table 7:** Effect of different loss functions on model performance.

Loss Function	P%	R%	F1%	mAP50%	mAP50-95%	Params/M	GFLOPs
DIOU	92.4	92.2	92.3	95.8	66.3	3.01	8.1
CIOU	92.9	91.6	92.2	95.8	66.5	3.01	8.1
EIOU	93.5	91.2	92.3	95.8	67.0	3.01	8.1
WIOU	92.8	91.8	92.3	96.0	66.8	3.01	8.1
NWD	94.1	93.0	93.5	96.6	66.9	3.01	8.1

### 4.4 Ablation experiments

As shown in **Table 8**, the F1, mAP50, and mAP50-95 of YOLOv8n on the test set are 92.3%, 95.5%, and 66.2%, respectively. Following the integration of Fasternet Block, the model’s parameter count and computational complexity were significantly reduced, accompanied by a minor decline in the F1 score and a slight enhancement in the mAP50 metric. After incorporating the EMA mechanism, only a slight increase in model parameters and computational effort was exchanged for a significant improvement in F1 score and mAP50-95 value, by 1.4% and 1.2%, respectively. The adoption of the DESCS-DH detection head further significantly reduced the model’s complexity, yielding a 0.1% increase in mAP50 value and a 0.2% reduction in the F1 score. With the introduction of the loss function NWD, the parameters and computational effort of the model did not change, but the F1, mAP50, and mAP50-95 improved by 0.9%, 0.2%, and 0.6%, respectively. Consequently, the model’s F1 score and mAP50 value were enhanced by 1.7% and 1.2%, respectively, compared to the baseline YOLOv8n model. Additionally, the model’s parameter count and computational effort were reduced by 44.8% and 39.5%, respectively.

**Table 8:** Results of ablation experiments.

Fasternet	EMA	DESCS- DH	NWD	P%	R%	F1%	mAP50%	mAP50-95%	Params/M	GFLOPs
×	×	×	×	93.2	91.3	92.3	95.5	66.2	3.01	8.1
✓	×	×	×	93.7	90.2	91.9	95.9	65.3	2.30	6.3
✓	✓	×	×	94.5	92.2	93.3	96.4	66.5	2.31	6.4
✓	✓	✓	×	93.8	92.5	93.1	96.5	66.3	1.66	4.9
✓	✓	✓	✓	94.5	93.6	94.0	96.7	66.9	1.66	4.9

The “✓” indicates that the model employs this module, while the “x” indicates that the model does not employ this module.

**Figure 8:**
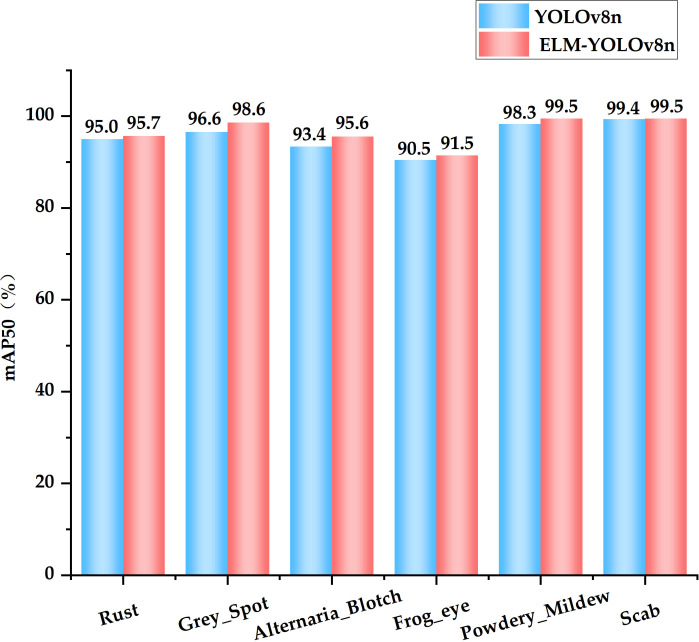
MAP50 values of YOLOv8n and ELM-YOLOv8n in the detection of six diseases.

**Figure 8** illustrates that the ELM-YOLOv8n model exhibits superior accuracy in identifying various apple diseases compared to the original YOLOv8n model, with the performance improvement being particularly pronounced for the detection of small target diseases. For example, the mAP50 indexes for detecting Grey Spot, Alternaria Blotch, and Frog Eye Leaf Spot, which are small target diseases, were improved by 2.0%, 2.2%, and 1.0%, respectively.

### 4.5 Comparison with other models

**Figure 9:**
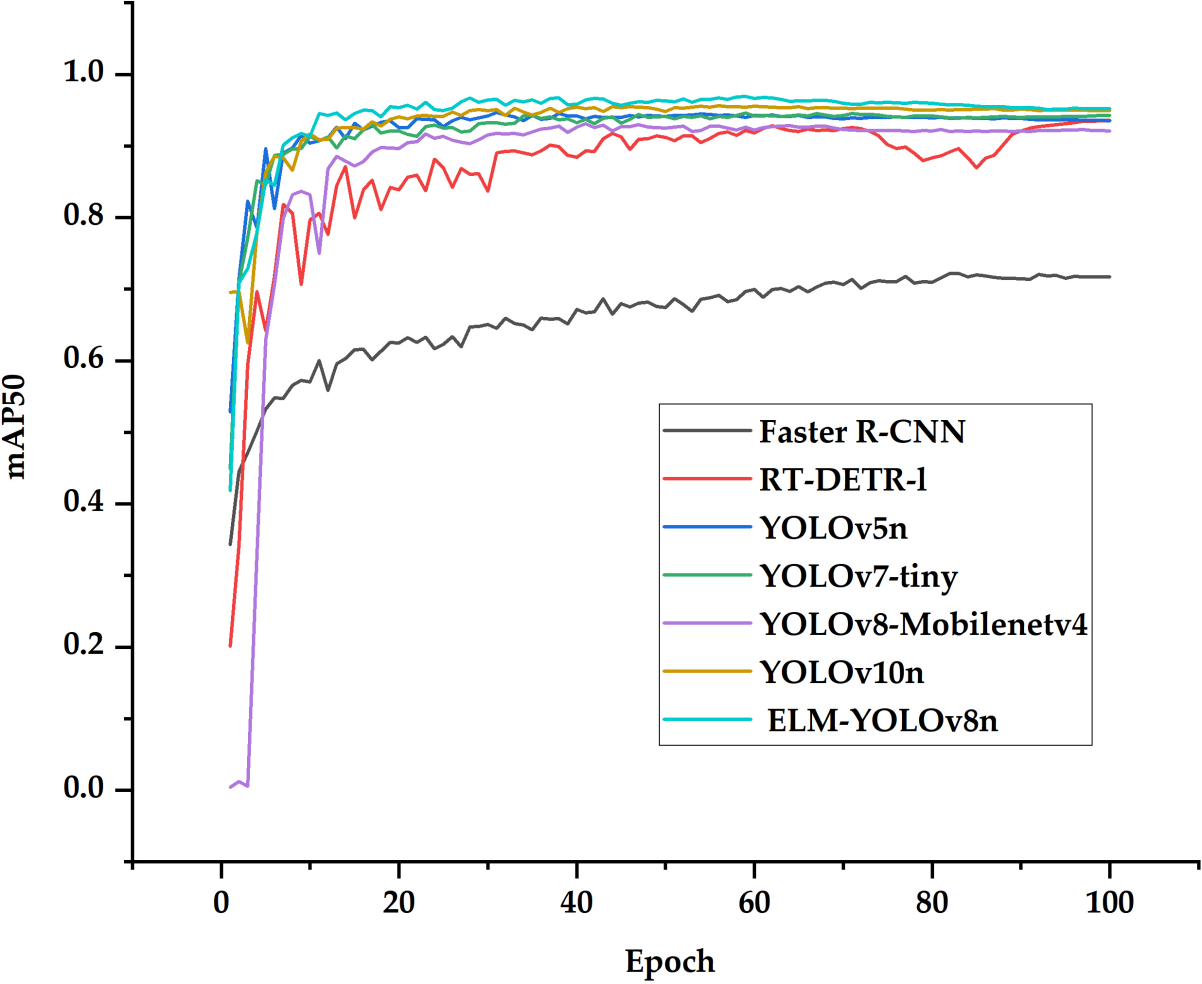
Changes in mAP50 during training of different models.

**Figure 10:**
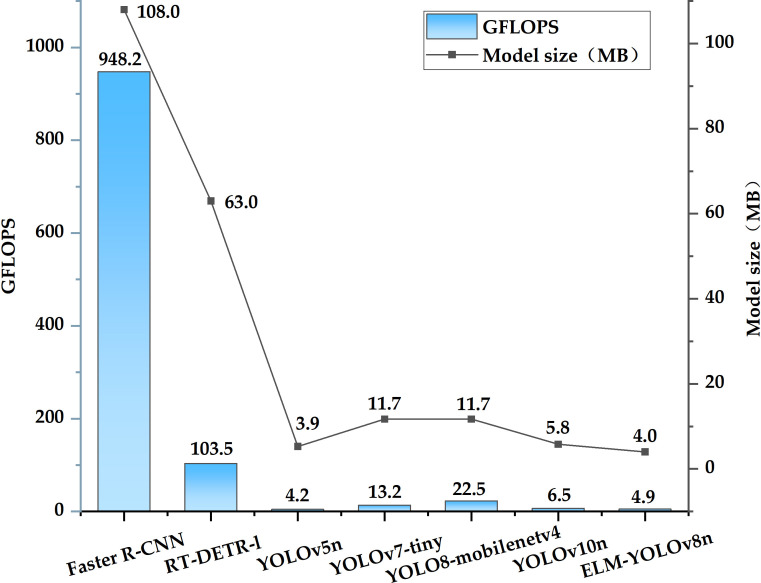
Comparison of computation and model size for different models.

**Table 9** presents the performance comparison of the seven object detection algorithms on the test set. ELM-YOLOv8n achieves the highest F1 and mAP50 scores of 94.0% and 96.7%, respectively, where mAP50 compares favorably with Faster R-CNN, RT-DETR-l, YOLOv5n, YOLOv7-tiny, YOLOv8-Mobilenetv4, and YOLOv10n by 24.2%, 1.8%, 2.1%, 0.9%, 3.3%, and 0.2%, respectively. **Figure 9** depicts the changes in mAP50 for various models throughout the training process. With respect to model complexity, ELM-YOLOv8n has the fewest parameters, totaling only 1.66M. Regarding computational and model size, as illustrated in **Figure 10**, Faster R-CNN exhibits the highest computation and largest model size, at 948.2 GFLOPs and 108.0 MB, respectively. In contrast, YOLOv5n has the smallest computation and model size, followed by the improved model, but the improved model’s F1, mAP50, and mAP50-95 values are 2.5%, 2.1%, and 3.1% higher than YOLOv5n, respectively.

**Table 9:** Performance comparison of different network models on test sets.

Model	P%	R%	F1%	mAP50%	mAP50- 95%	Params/M	GFLOPs
Faster R-CNN	70.2	74.5	72.3	72.5	42.1	28.33	948.2
RT-DETR-l	91.7	91.8	91.7	94.9	65.0	32.0	103.5
YOLOv5n	91.9	91.1	91.5	94.6	63.8	1.77	4.2
YOLOv7-tiny	92.8	91.7	92.2	95.8	65.2	6.02	13.2
YOLOv8-Mobilenetv4	92.1	90.0	91.0	93.4	63.3	5.70	22.5
YOLOv10n	93.5	93.0	93.2	96.5	67.7	2.27	6.5
ELM-YOLOv8n	94.5	93.6	94.0	96.7	66.9	1.66	4.9

### 4.6 Generalization experiment

**Figure 11:**
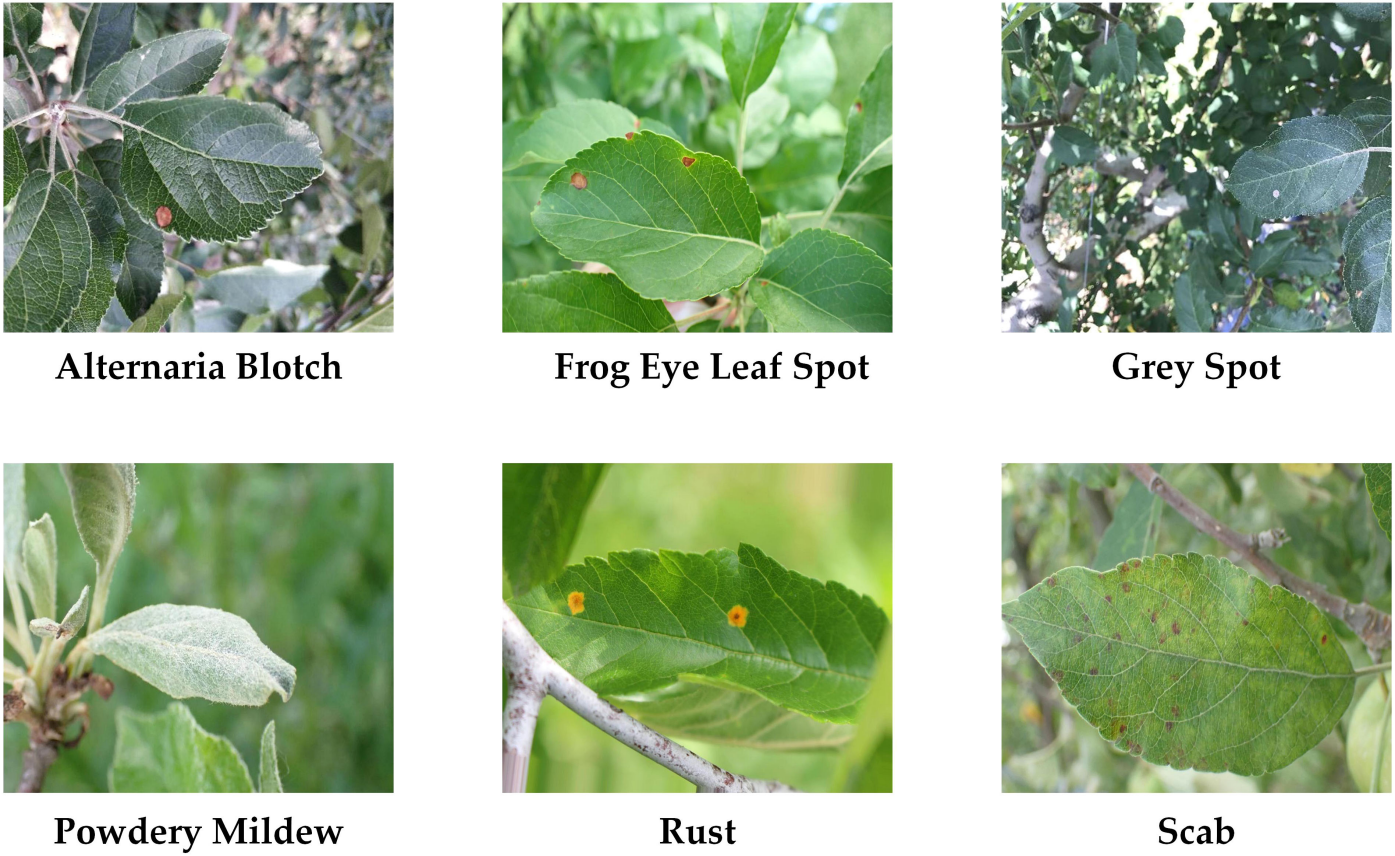
Example of Apple-leaf2 dataset.

To evaluate the model’s adaptability to new data and its performance in various environments and scenarios, we conducted generalization experiment. A collection of 823 apple disease images from different scenarios were selected to be collected under different light, weather and geographic locations, with 755 images from field scenarios and 68 images from simple scenarios to simulate a variety of situations in the real world, thus testing the stability of the model under these conditions. Images were selected to cover a wide range of disease types including Rust, Scab, Grey Spot, Frog Eye Leaf Spot, Powdery Mildew, and Alternaria Blotch, naming this dataset Apple-leaf2.The example of the dataset is illustrated in **Figure 11**.

Experimental results show that the model can work efficiently in simple scenarios as well as remain robust in complex scenarios. As shown in **Table 10**, the ELM-YOLOv8n model exhibits an enhanced F1 score of 0.8% and an mAP50-95 value improvement of 1.1%, relative to the YOLOv8n. Concurrently, the parameter count and computational complexity are diminished by 44.8% and 39.5%, respectively. These results suggest that the improved model has strong detection capabilities for leaf diseases in multifactorial complex scenarios and is more suited for deployment on mobile devices.

**Table 10:** YOLOv8n and ELM-YOLOv8n detection results on Apple-leaf2 dataset.

Dataset	Model	P%	R%	F1%	mAP50%	mAP50- 95%	Params/M	GFLOPs
Apple-leaf2	YOLOv8n	85.5	94.5	89.8	94.8	57.8	3.01	8.1
	ELM-YOLOv8n	85.6	96.3	90.6	95.6	58.9	1.66	4.9

**Figure 12:**
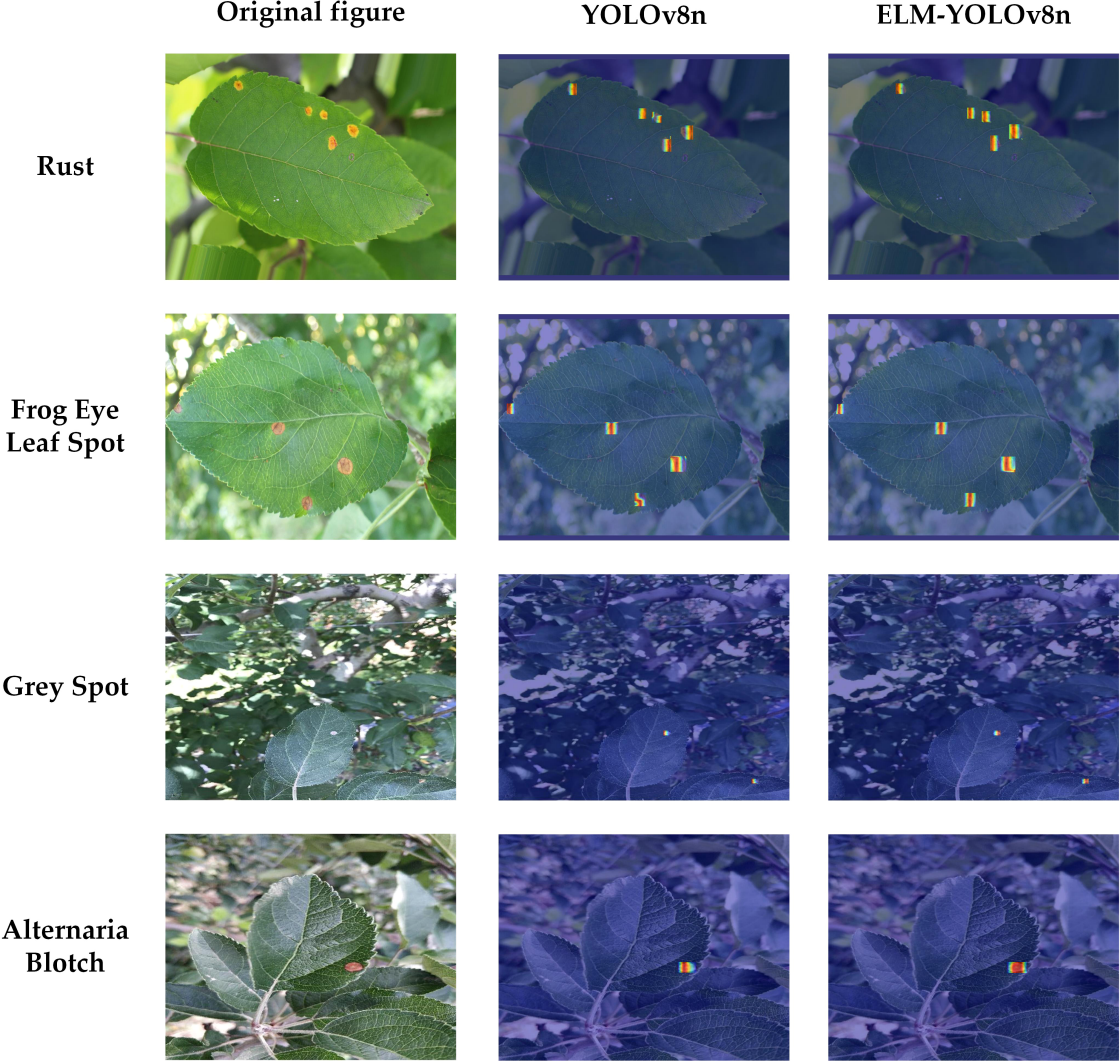
Comparison of YOLOv8n and ELM-YOLOv8n heat maps on selected test sets.

To intuitively illustrate the interpretability of the model’s decisions, we utilize XGrad-CAM to visualize the model’s predictions. This method involves computing the gradient of the feature map in the convolutional neural network relative to the target category, combining these gradients with the feature map values to obtain importance scores, and normalizing the result to create a heat map that highlights the critical regions in the image for decision-making. **Figure 12** displays the heat maps comparison between YOLOv8n and ELM-YOLOv8n on selected test sets. The heat maps generated by ELM-YOLOv8n reveal that the predicted targets exhibit darker colors, signifying that the model allocates greater attention to regions of interest within the target image during the prediction.

## 5 Discussion

Current apple disease detection algorithms confront various challenges, including large model parameters, high computational complexity, and long inference times (Ma et al., 2024). To effectively address these issues and achieve accurate detection of apple leaf diseases, this study introduces the Fasternet Block and proposes a detail-enhanced shared convolutional scaling detection head (DESCS-DH) for constructing a lightweight, real-time object detection model. Incorporating the Fasternet Block into C2f reduced the model’s parameters and computational requirements by 23.5% and 22.2%, respectively. The F1 score slightly decreased by 0.4%, while the mAP50 increased by 0.4%. This can be attributed to PConv in Fasternet, which convolves only a subset of input channels, significantly reducing model complexity and storage space needs. This allows the Fasternet Block to effectively manage the number of model parameters with minimal performance degradation. Utilizing DESCS-DH as the detection head for YOLOV8, the model’s mAP50 is enhanced by 0.8% while simultaneously decreasing model complexity. DESCS-DH is an efficient and lightweight detection head that boasts several advantages, including the use of shared convolutions for parameter sharing, which drastically decreases the number of parameters. In the task of apple leaf disease detection, critical feature information of the target, such as disease edges and textures, is often found in image details. Standard convolution operations may overlook these subtle yet vital details; however, DEConv within DESCS-DH effectively captures them. When combined with re-parameterization techniques, DEConv optimizes the convolution operation without increasing the model’s computational load, thereby enhancing the model’s responsiveness to input data. In real-world disease detection scenarios, the target sizes vary significantly. DESCS-DH can adaptively modify its internal parameters, ensuring that the detection head maintains stable performance across a range of target sizes.

In the recognition of small target diseases on apple leaves within complex backgrounds, the primary challenges are the small size of target objects, their subtle features, and their susceptibility to interference from background noise, lighting variations, leaf occlusion, and similar textures. These challenges compound the difficulty of detection, thereby diminishing recognition accuracy (Shao et al., 2024; Wang et al., 2024a). Incorporating the EMA mechanism into YOLOv8n enhances the mAP50 by 0.8%. The EMA mechanism constructs local cross-channel interactions within each parallel sub-network without reducing channel dimensions. Through a cross-space learning approach, it fuses the output feature maps of the two parallel sub-networks and generates better pixel-level attention for high-level feature maps. Thus, the model can focus on the disease area and reduce the influence of disturbing factors, even in the presence of light variations or background noise. Spatial attention weights, processed by the sigmoid function, capture pairwise pixel relationships, facilitating the model’s comprehension of structural information within the diseased area and enabling the recognition of minute disease features through pixel interactions, even when such features are not readily apparent. In the detection and localization of small target lesions, they occupy fewer pixels in the image, which makes it difficult for the model to accurately determine the category and location of the target, while the NWD (Normalized Wasserstein Distance) loss function is sensitive to the size and distance of the target. Utilizing the NWD as a loss function for YOLOV8, the model’s F1 score is enhanced by 1.2%, and the mAP50 value is incremented by 1.1%.

Apple orchards usually have a large area, and traditional artificial disease detection methods are not only extremely inefficient, but also difficult to achieve comprehensive and timely monitoring. The enhanced lightweight model proposed in this study has significant features such as high precision, low computational complexity and small model size, which makes it very suitable for deployment on mobile devices with limited resources. In the next research, we plan to deploy the model on the UAV. By integrating edge computing devices on the UAV, the model can process the collected image data in real time during UAV flights, timely and accurately identify diseases in the orchard, and provide support for the disease prevention and control of the orchard. The UAV can automatically fly according to the preset route and complete the scanning of the entire orchard in a short time, which greatly improves the detection efficiency.

The ideal UAV platform should have the following characteristics: sufficient computing power(GPU over 2GB of VRAM), a high-resolution camera, equipped with a Global Positioning System (GPS), automatic flight control, wireless communication systems, multiple sensors, and other functional components, preferably a multi-rotor UAV (Chen et al., 2021). However, the accuracy of the model is affected by the distance of the drone’s image capture and the flight speed. Based on theoretical analysis, in order to ensure a certain field of view and obtain a clear image of the leaf, the appropriate flight altitude should be maintained 3-5 meters (Hou et al., 2023), which can meet the needs of most disease detection. With the improvement of camera resolution, the height can be increased to more than 5 meters. The appropriate flight speed should be set 1.5-3 m/s. By adjusting the shooting parameters, most common diseases can be accurately recognized. As the flight speed increases, the recognition accuracy of the model will decline, and there may be a small amount of missed detection for some extremely subtle disease features, such as the early mild symptoms of apple powdery mildew. In addition, the present model has some limitations in dealing with the presence of multiple diseases in a leaf, because the training data primarily target the case of a single disease. Therefore, the model still requires further optimization and improvement for the identification of multiple diseases. We will work on these challenges in our future work to enhance the comprehensive performance and application scope of the model.

## 6 Conclusions

To achieve real-time detection of apple leaf diseases in complex environments, this study introduces an enhanced lightweight model, ELM-YOLOv8n.In order to overcome the challenge of inadequate feature extraction from small targets in complex backgrounds, we incorporate the Efficient Multi-Scale Attention (EMA), which captures both channel and spatial information, enhancing feature representation without a significant increase in parameters or computational cost. To decrease the parameter count and computational load, the Fasternet Block is integrated into the C2f module of the backbone and neck, substantially reducing the model’s complexity. In order to make the network more lightweight and better extract the edge information of the disease, we design the Detail Enhanced Shared Convolutional Scaling Detection Head (DESCS-DH), which can maintain the high accuracy with fewer parameters and computation. The detection head can adaptively modify its internal parameters in response to the input feature map size, ensuring optimal operating parameters for multi-scale targets, thereby enhancing resilience to environmental changes and interference. Lastly, utilizing the NWD loss function enhances the model’s precision in localizing and identifying small targets, which further enhances the model’s recognition accuracy.

The experimental results indicate that the ELM-YOLOv8n algorithm performs well in terms of computational resource consumption while maintaining excellent detection accuracy, which fully meets the demand for real-time processing. Compared to other current models, this approach not only significantly improves the detection accuracy, but also drastically reduces the demand for computing platform resources, making it more feasible to be deployed on resource-constrained devices. Future research will concentrate on enhancing the model’s robustness across various environmental conditions and on optimizing its capability to detect and process a broader range of crop diseases. The optimized model is poised for successful application in resource-limited embedded detection systems, with the algorithm set to undergo further refinement to guarantee its efficiency and reliability in practical settings.

## Data Availability

The original contributions presented in the study are included in the article/supplementary material. Further inquiries can be directed to the corresponding author.
